# Dual-Probe Transcranial Full-Waveform Inversion: A Brain Phantom Feasibility Study

**DOI:** 10.1016/j.ultrasmedbio.2023.06.001

**Published:** 2023-07-18

**Authors:** Thomas Caradoc Robins, Carlos Cueto, Javier Cudeiro, Oscar Bates, Oscar Calderon Agudo, George Strong, Lluis Guasch, Michael Warner, Meng-Xing Tang

**Affiliations:** a Department of Bioengineering, Faculty of Engineering, Imperial College London, London, UK; b Department of Earth Science and Engineering, Faculty of Engineering, Imperial College London, London, UK

**Keywords:** Ultrasound tomography, Brain imaging, Full-waveform inversion, Brain phantom

## Abstract

**Objective:**

Despite being a low-cost, portable and safe medical imaging technique, transcranial ultrasound imaging is not used widely in adults because of the severe degradation and distortion of signals caused by the skull. Full-waveform inversion (FWI) has recently been found to have potential as an effective method for transcranial ultrasound tomography to obtain high-quality, subwavelength-resolution acoustic models of the brain using low-frequency ultrasound data. In this study is the first demonstration of this method in recovering a high-resolution 2-D reconstruction of a brain and skull ultrasound imaging phantom using experimentally acquired data.

**Methods:**

A 2:5 scale brain phantom encased within a 3-D-printed skull-mimicking layer was created to simulate a clinical transcranial imaging target. To obtain tomographic ultrasound data on the brain and skull phantom, a tomographic ultrasound acquisition system was designed and implemented using commercially available low-frequency cardiac probes. FWI reconstructions of the brain and skull phantom were performed using the acquired tomographic data and were compared with corresponding synthetic reconstructions. This comparison was used to evaluate the feasibility of the proposed imaging system when employing different transducer array configurations.

**Results:**

We demonstrate the successful FWI reconstruction of the brain phantom within the skull mimic from experimentally acquired tomographic ultrasound data. To mitigate the effects of the skull-mimicking material, a reflection-matching algorithm was applied to model the morphology of the skull layer prior to performing the inversion.

**Conclusion:**

The findings of this study provide a promising step toward the clinical use of FWI for transcranial ultrasound imaging in adults.

## Introduction

The accessibility, portability, inherent safety and low cost of ultrasound imaging devices have made medical ultrasound one of the most widely used non-invasive imaging modalities. Consequently, transcranial sonography has become an indispensable imaging modality for the routine care of newborns. Despite these benefits, high-resolution ultrasound imaging methods cannot currently image adult human brains effectively. This is owing to the severe degradation of high-frequency ultrasound signals used in conventional transcranial sonography (5−12 MHz) when developed skull tissue is present, which is observed in patients older than 6 mo [1]. Furthermore, adult skull tissue also produces distortions and scattering of transcranial ultrasonic waves. Imaging with low-frequency ultrasound (<1.00 MHz) can overcome the effects of skull attenuation on ultrasonic waves [2], allowing for the acquisition of higher signal-to-noise ratio (SNR) ultrasound data. Previous studies have applied this approach to perform structural imaging of the brain using conventional ultrasound imaging methods, such as pulse−echo B-mode [3]. Alternatively, the blood flow in major intracranial arteries can be measured by imaging in the range 1.00−3.00 MHz through thin sections of bone located at the temples of the skull [4]. Previous studies have explored the use of dual probes to perform brain imaging through these bone windows, such as the study by Vignon et al. [5], in which linear arrays were positioned at both temples of an ex vivo human skull to image a brain phantom. Acoustic signals transmitted between these opposing arrays were used to correct for the impact of skull aberrations and to estimate the frequency-dependent attenuation of ultrasonic waves. A similar method proposed by Lindsey et al. [6] used dual probes with 2-D arrays to correct for aberrations and image a common volume within a brain phantom transcranially to perform 3-D colour flow imaging. However, imaging at these lower frequencies comes at the expense of spatial resolution, which prevents the acquisition of high-resolution, diagnostically useful images. Furthermore, these methods are limited to imaging only those portions of the brain visible through the bone windows, which limits the potential applications of transcranial ultrasound.

Recently, full-waveform inversion (FWI) has been proposed as a promising ultrasound tomography method for recovering high-resolution acoustic models of the full brain at sub-millimeter resolutions [7,8]. FWI is a data-fitting technique originally developed for seismic exploration to improve models of the sub-surface [9]. The method aims to iteratively refine an acoustic model so that the misfit between observed and numerically generated data sets is minimised.

**Figure 1:**
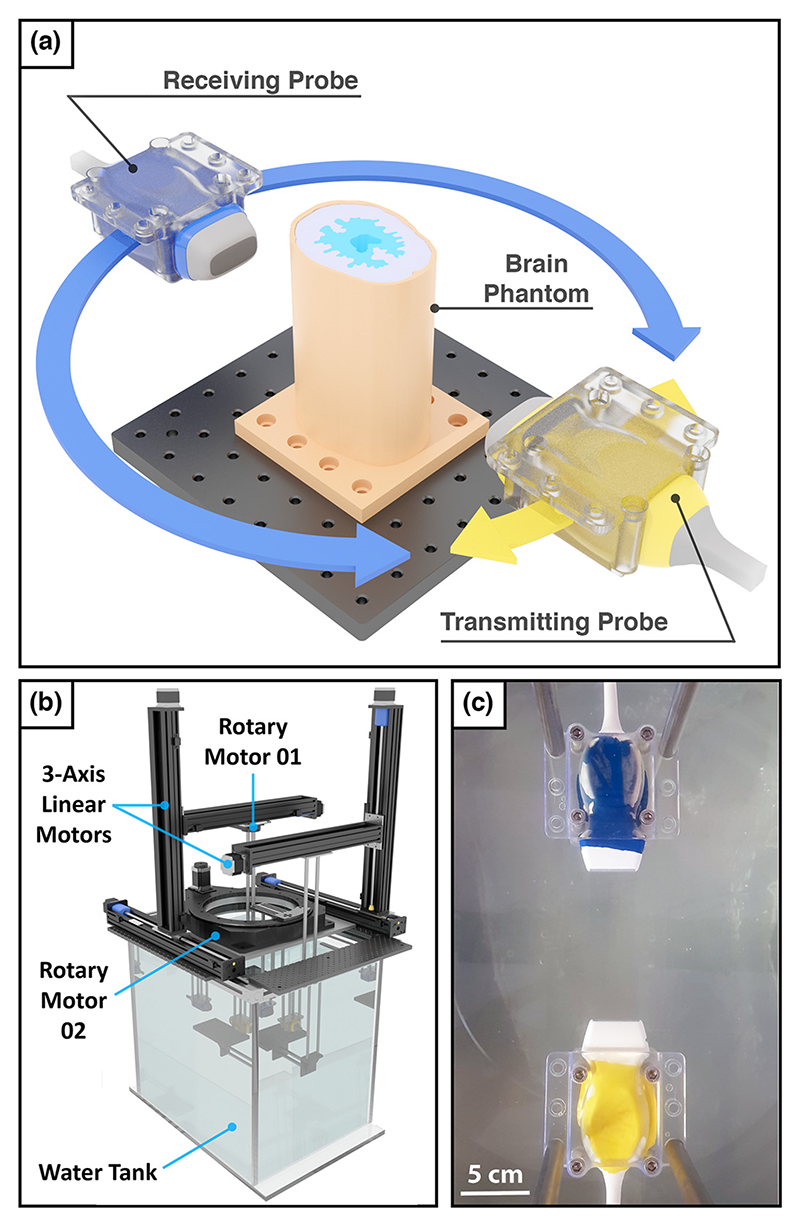
Dual-probe ultrasound tomography experimental setup. A pair of medical probes are translated independently about a 2.5-D brain and skull-mimicking phantom to acquire transcranial ultrasound tomography data.

In this study, we demonstrate for the first time the feasibility of applying FWI to reconstruct a brain-tissue phantom surrounded by a skull-mimicking layer. To achieve this, we designed an ultrasound computed tomography (USCT) acquisition system using a pair of off-the-shelf clinical cardiac probes (P4-1 ATL, Philips Healthcare, Bothell, WA, USA) that could be positioned to perform imaging tasks using a combination of linear and rotary motors, as seen in Figure 1. While it has been reported that 3-D FWI is required to image complex geometries such as the head because of out-of-plane effects [10], in this study, we have chosen to constrain the dimensionality of the problem to two dimensions. This was done to simplify the acquisition of USCT data and to reduce computational costs when running FWI. Therefore, we designed a brain and skull-mimicking phantom that consists of a 2-D brain and skull image extended along the vertical axis to provide the 2.5-D imaging phantom seen in Figure 1a. This phantom was designed to provide an imaging target that mimics the skull and brain tissue we would expect to encounter when imaging patients in a clinical setting.

## Methods

### Full-waveform inversion

Full-wave inversion is an iterative image-reconstruction technique that aims to ptimize the misfit between observed data **d** found experimentally and synthetic wave-field data **p** generated numerically by solving the wave equation for a model of acoustic properties **m**. Given that the wave equation is a non-linear function with respect to the model parameters, it cannot be directly inverted. FWI instead poses this problem as an iterative local-gradient descent least-squares localisation to ptimize the following objective function f with respect to **m**:

(1) $$f(\text{m})=\frac{1}{2}∥\delta \text{d}{∥}_{2}^{2}=\frac{1}{2}∥\text{d}-\text{p}{∥}_{2}^{2}$$

Here, *δ***d** is the residual between the observed and predicted signal data. At each iteration, we seek to solve this problem by determining the direction of steepest descent on the hyper-surface defined by f. From an initial starting model m_0_, this direction is taken to calculate model updates that will tend towards a model that minimizes the difference between predicted signal data and the observed signal data. To find this direction, the partial derivative of f with respect to **m** is computed by solving the adjoint-state problem [7]:

(2) $$\frac{\partial f}{\partial \text{m}}={\left[\frac{\partial \text{p}}{\partial \text{m}}\right]}^{T}\delta \text{d}$$

To solve the derivative of **p** with respect to **m**, we consider the wave equation problem in the discrete form as

(3) A(m)u=s

where **A** is the discrete wave equation, **u** is the pressure wavefield and s represents the source. Given that s is not dependent on **m**, differentiating eqn (3) with respect to **m** gives

(4) $$\text{A}\frac{\partial \text{u}}{\partial \text{m}}\text{+}\frac{\partial \text{A}}{\partial \text{m}}\text{u}=0$$

Assuming eqn (4) has a unique solution, we invert **A** to yield

(5) $$\frac{\partial \text{u}}{\partial \text{m}}=-{\text{A}}^{-1}\frac{\partial \text{A}}{\partial \text{m}}\text{u}$$

While **u** represents the wavefield, **p** represents wavefield data sampled only at the receiving transducer element positions. This is performed by sampling **u** at positions defined by the matrix **R** such that **p = Ru**. Consequently, the differential of **p** with respect to **m** is given by

(6) $$\frac{\partial \text{p}}{\partial \text{m}}=-{\text{RA}}^{-1}\frac{\partial \text{A}}{\partial \text{m}}\text{u}$$

Given that **R** is not dependent on **m**, this gives the final equation for the gradient of f as

(7) $$\frac{\partial f}{\partial \text{m}}=-{\text{u}}^{T}\frac{\partial {\text{A}}^{T}}{\partial \text{m}}{\text{A}}^{-T}{\text{R}}^{T}\delta \text{d}$$

In summary, given the model of the latest iteration **m**_0_, the synthetic wavefield **u** can be generated by approximating the solution of the acoustic wave equation using a high-order finite-difference forward model. This wavefield is then sampled at the transducer receiver positions to give the predicted signal data **p** by applying the restriction matrix **R**. By use of eqn (7), the gradient of the steepest descent is then calculated by first computing the residual *δ***d** between **p** and **d**. The backpropagation of the wavefield is then computed by injecting the residual back into the model at the transducer receiver positions. The resulting time-reversed wavefield is then scaled using the differential of the wave equation operator **A** with respect to **m**. Finally, the zero lag of the cross-correlation of the time-reversed wavefield and the initial wavefield **u** is calculated for every point in **m** in the time domain. This provides *δ***m**, the model update which applies a small perturbation of the model towards the direction that will ptimize f. Given that this method will tend towards the direction of steepest descent, this FWI method is susceptible to arriving at local minima should the initial starting conditions not place the inversion solution on a gradient that tends towards the global minimum. It is therefore important that the initial model conditions are sufficiently close to the neighbourhood of the global minimum to avoid erroneous reconstructions when running FWI. This is discussed in more detail in the phantom scaling section of this study when considering the cycle-skipping phenomena, and examples of these erroneous reconstructions caused by cycle skipping are discussed under Ring Array Brain Phantom Reconstruction Results.

### 2.5-D brain and skull-mimicking phantom

The 2.5-D brain and skull-mimicking phantom designed for this study is illustrated in Figure 1a. This phantom consisted of a skull-mimicking layer and a multilayered brain model with irregular features. In this section, we discuss the design, fabrication and properties of this phantom.

### Skull-mimicking layer

**Figure 2:**
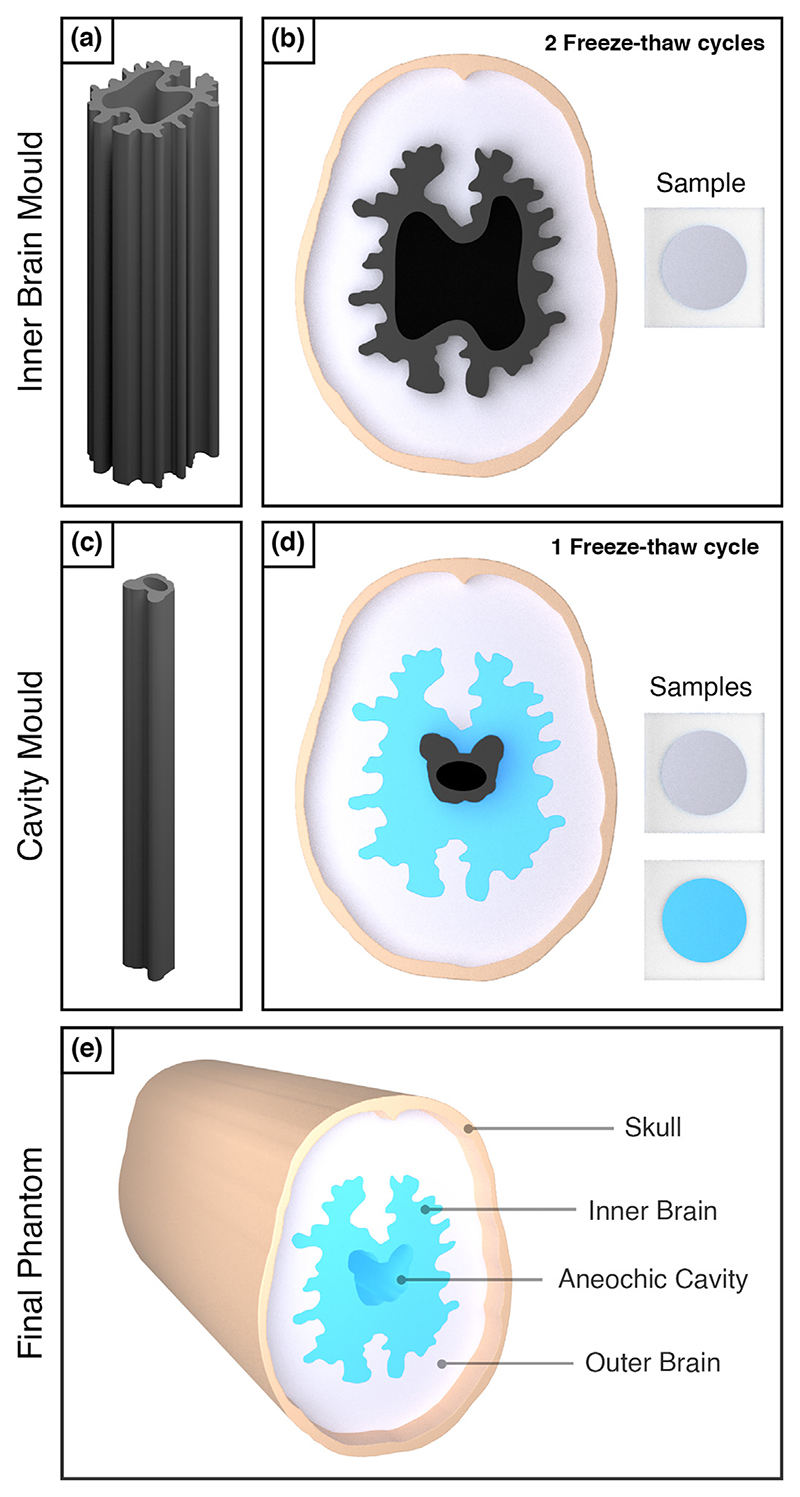
Fabrication of the 2.5-D brain and skull phantom. (a) Three-dimensional printed resin mould for providing the irregular boundary between the inner and outer brain layers. (b) The 2.5-D phantom and outer brain tissue mimic sample during the outer brain PVA cryogel conditioning step. (c) Three-dimensional printed resin mould for providing the boundary of the central cavity of the brain model. (d) The 2.5-D phantom and brain tissue mimic samples during the final PVA cryogel conditioning step. (e) Three-dimensional render of the finished skull and brain 2.5-D phantom.

We designed a skull-mimicking layer to model the morphology and acoustic properties of a real skull. To achieve this, we used a 2-D axial cross-section of a skull sampled from the numerical MIDA head model, which provided an anatomically accurate head model with a spatial resolution of 5.00 × 10^−1^ mm [11]. This skull cross-section was extruded to form a continuous shell to surround the brain-mimicking phantom, as illustrated in Figure 2e. To select an appropriate skull-mimicking material, we considered the composition of skulls and the variation in measured speed-of-sound (SoS) values between different skull tissues. Skull tissue typically consists of spongy cancellous skull tissue surrounded by outer and inner cortical skull tissue layers [12]. Acoustic measurements have revealed that these different skull tissues have distinct SoS values, as seen in Table 1 using skull SoS measurements provided by the IT’IS tissue property database, Version 4.0 [13]. To determine an SoS range in which to select the skull-mimicking material, we used a simplified model of a typical cross-section of an adult human skull, based on mean thickness measurements provided by Lillie et al. [12]. This skull cross-section model was given a total thickness of 5.00 mm and consisted of an outer cortical layer (2.13 mm), a central cancellous layer (1.90 mm) and an inner cortical layer (1.47 mm). By optimised the measured values of the SoS for each skull layer, the time taken for an ultrasonic wave to pass through the skull model was determined to be 2.21 ± 0.28 *μ*s. Therefore, on the basis of this calculation, a skull-mimicking layer consisting of a single material should ideally have a SoS in the range of 2526.95 ± 320.00 m/s to have an SoS comparable to that of this skull composite model.

Previous studies have found that skull tissue porosity affects imaging performance, particularly at high angles of incidence [14]. This microstructure scatters ultrasound waves and would need to be introduced to model the propagation of real-world ultrasonic waves accurately. However, for this study, we attempted to image without introducing porosity to the 3-D-printed skull layer so we can first assess the suitability of conventional ultrasound devices and our FWI method to recover the brain model while imaging through high SoS skull-mimicking material.

Skull-mimicking materials previously investigated for creating realistic skull phantoms include Araldite 1302 and VeroBlack [15]. Araldite 1302, an epoxy resin, can be used to cast anatomically accurate skull phantoms by being poured into skull-shaped moulds. It has a measured SoS of 3008 ± 220 m/s, making it comparable to the SoS of real cortical skull tissue [15]. Alternatively, VeroBlack, a rigid thermoset acrylic resin, is ideal for creating 3-D-printed skull phantoms based on CT data. The 3-D printer method produced more accurate and consistent skull models than the Araldite casting method [15]. Furthermore, VeroBlack was measured to have an SoS of 2495 ± 8 m/s, making it a much closer match to the previously calculated ideal skull-mimicking SoS range. Based on these findings, we opted for a 3-D printing approach for fabricating the skull layer of the skull and brain-mimicking phantom. To produce accurate and repeatable skull layer models, we used stereolithography, a 3-D printing method known for producing highly accurate, isotropic and watertight components [16]. The skull-mimicking material we selected for the skull layer model was a photopolymer resin (Clear Resin, Formlabs Inc., Somerville, MA, US). As outlined in Table 1, acoustic measurements of a regular 5.00 mm thick 3-D-printed block of Formlabs Clear Resin gave an SoS of 2545 ± 11 m/s, placing it within the ideal skull-mimicking material SoS range. This value is comparable to measurements by Bakaric et al. [17], which gave a group velocity and density for the Formlabs clear 3-D-printed photopolymer of 2591 m/s and 1178 kg/m^3^, respectively. Here, the group velocity is given as the speed at which the envelope of the transmitted signal propagates across the bone-mimicking material. This suggests that the SoS range of the 3-D-printed skull-mimicking layer created using Formlabs Clear Resin is a suitable match to the ideal SoS range of real skull tissue and is capable of providing accurate and repeatable results in our experiments.

### Brain-mimicking model

The brain tissue-mimicking phantom was cast using 3-D-printed resin moulds to create layers of polyvinyl alcohol (PVA) cryogel with contrasting SoS values within the range 1500−1520 m/s. As seen in the 3-D render of the completed 2.5-D phantom in Figure 2e, these layers include an outer white matter layer (~1500 m/s), an inner grey matter layer (~1520 m/s) and a central water-filled cavity. When combined, these different layers form a brain model with complex features and three regions with contrasting SoS values to be recovered using FWI.

We fabricated the PVA cryogel brain tissue mimics using an approach similar to the tissue-fabrication protocol proposed by Chee et al. [18]. The first step of this method was to heat a PVA cryogel solution consisting of 10% PVA powder and 90% distilled water at 90°C for 1 h. This solution was then cooled to 40°C and set using 24-h freeze−thaw cycles to increase the amount of PVA localisation. Each freeze−thaw cycle consisted of storing the tissue mimic for 12 h in a freezer at −20°C, followed by 12 h at room temperature. Two contrasting brain-mimicking tissues were created by varying the number of these freeze−thaw cycles, as indicated by the *blue and white regions* of the brain model seen in Figure 2.

To introduce complex, irregular geometry into the brain model, we 3-D-printed the resin brain moulds shown in Figure 2a and 2c to cast the different brain layers. These included an outer mould designed to cast the outer brain layer and an inner mould to shape the inner brain layer. To create the outer brain layer, the PVA cryogel solution was poured into the cavity between the skull layer and the outer mould when placed in the centre of the phantom, as illustrated in Figure 2b. This outer brain layer was then partially set using two freeze−thaw cycles. The outer mould was then removed and replaced by the inner mould, creating a new cavity for the inner brain layer. A new batch of PVA cryogel solution was poured into this cavity, as illustrated in Figure 2d (blue food colouring was added to this solution to indicate the different brain layers). The phantom then underwent one additional freeze−thaw cycle to set the inner brain layer. Therefore, the outer layer underwent a total of three cycles, while the inner layer underwent one cycle. The testing samples illustrated in Figure 2d were taken at each PVA cryogel pour and were subjected to the same conditions as their corresponding PVA cryogel brain layers. These samples could then be measured to provide estimates for the SoS values of each brain tissue mimic. The results of these measurements are provided in Table 1.

### 2.5-D brain and skull phantom scaling

Acquiring USCT data with sufficiently low-frequency content can mitigate the phenomenon of cycle skipping, which is well documented in geophysical FWI applications and occurs when the predicted and observed wavefield data are more than 90° out of phase [19]. Cycle skipping can lead the localisation problem to fall into local minima, resulting in reconstructions with inaccurate information. In the in silico FWI brain imaging study discussed by Guasch et al. [8], USCT with a bandwidth of 0.10−0.85 MHz was chosen to mitigate the effects of cycle skipping when SoS skull tissue was present. However, medical ultrasound probes are typically not designed to image at sub-megahertz frequencies, making them ill-suited for acquiring data in this range. For this study, we selected a pair of cardiac probes (P4-1 ATL) with a frequency range of 0.55−3.50 MHz, making them among the lowest-frequency medical transducers currently available. To account for these different band-widths, we fabricated a phantom at a 2:5 scale relative to the original MIDA model. The resulting skull model had a width of 60.54 mm, a length of 78.87 mm, a height of 130.00 mm and a mean thickness of 2.24 ± 0.48 mm.

### Dual-probe data acquisition system

The experimental USCT data sets for this study were acquired using a dual-probe USCT imaging rig. The rig consisted of a pair of P4-1 cardiac probes, an ultrafast ultrasound research acquisition system with two 128-channel ports (256 Vantage System, Verasonics, Kirkland, WA, US) and a combination of linear and rotary motors. The acquisitions were performed in the centre of a large 0.7 × 0.6 × 0.6 m^3^ Perspex water tank to minimize the detection of strong reflections from the water surface and the sides of the tank. The full dual-probe USCT system can be seen in Figure 1b. While connected to the Vantage acquisition system, these probes can be controlled as a single 192-element array. For a transmission event when any one element is selected as a source, the elements of both probes could be assigned as receivers to allow for both transmitted and reflected ultrasonic waves to be sampled at different positions. When imaging, both probes are positioned to be facing each other, co-planar and with their elements aligned with the vertical y-axis. The resulting imaging region between these probes can be seen in Figure 1c. This region is defined as lying on the x−z plane, while all space-dependent fields are considered invariant with respect to y. The transducer elements are much greater in height than in width (height ~ 13.00 mm, width ≈ 2.45 × 10^−1^ mm). Consequently, these elements can be considered infinitely long sources relative to the x−z imaging plane [20]. Furthermore, as the width of the P4-1 elements is less than the smallest wavelength observed in the USCT data when running FWI (*λ*_min_ = 9.25 × 10^−1^ mm), these elements can be considered point sources and receivers within the imaging plane.

### Dual-probe imaging sequence

Imaging sequences using the dual-probe system involve the independent positioning of the probes for transmission and reception of ultrasonic waves at different positions using a combination of motors. To improve the SNR of signal data, each acquisition for a given source element was taken as the mean of eight sequential transmission events with a delay of 6.0 × 10^−4^ s between shots. Imaging with just one set of stationary probe positions was limited as the aperture of a P4-1 probe is only 28.32 mm. Therefore, we introduced motor steps to increase the number of probe positions and orientations during imaging sequences, which effectively increased the imaging aperture. Two different transducer array configurations were investigated: a linear array configuration using 1-D linear motor steps to form two parallel rows of probe positions and a ring array configuration of probe positions.

We acquired a corresponding watershot data set for each imaging experiment that used the same imaging sequence but without the imaging target in place. The resulting watershot data sets contained only the direct transmission of ultrasonic waves between the source and receiver transducer elements. As such, these watershot data provided valuable information to calibrate the relative positions of transducers in the dual-probe system and model the wavefields generated by each source element.

### Optimising full-waveform inversion parameters

Because of the nature of the data acquisition method, discrepancies may arise between the expected and actual positions of the probes during imaging sequences. Furthermore, the source wavelets generated by each element of the probes are initially unknown, and the water velocity may vary between experiments because of environmental factors such as temperature. As such, we initially treat the water velocity, transducer positions and source wavelets as unknown parameters that need to be estimated. These are needed to accurately model transmission events during imaging sequences while running FWI. In this section, we discuss how we estimated these acoustic modelling parameters.

### Water velocity estimation

We estimated the water velocity at the time each imaging experiment took place by determining the rate of change in travel time between source−receiver pairs as a function of distance. We achieved this by measuring the travel time through water between the probes at different distances using a linear stepper motor. We let t_0_ be the initial travel time between two aligned and opposing probe elements, t_n_ be the travel time for the same source−receiver pair after n linear motor steps of size x in the axial direction and N be the total number of steps. An estimate for the water velocity c is then given by

(8) $$c=\frac{\sum_{n=1}^{N}\left(\frac{nx}{t_{n}-t_{0}}\right)}{N}$$

In practice, the travel time differences were found by cross-correlating each new trace with the initial watershot trace and taking the position of the resulting maxima.

### Transducer position calibration

To calibrate transducer element positions, we developed a time-of-flight (TOF) element-localisation method based on the USCT calibration approach proposed by Filipik et al. [21]. The TOF of a watershot trace from a source at point [x_s_, y_s_, z_s_] to a receiver at point [x_r_, y_r_, z_r_] respectively) can be calculated as the sum of the travel time to traverse the distance d_sr_ through water, with an acoustic velocity c, and the system delay *τ* introduced by the acquisition system. This TOF value can be defined as

(9) $$TOF_{sr}=\frac{d_{sr}}{c}+\tau$$

Where

(10) $$d_{\text{sr}}=\sqrt{{\left(x_{\text{s}}-x_{\text{r}}\right)}^{2}+{\left(y_{\text{s}}-y_{\text{r}}\right)}^{2}+{\left(z_{\text{s}}-z_{\text{r}}\right)}^{2}}$$

The value for *τ* was considered to be constant across all traces as all data were acquired using a pair of identical P4-1 probes. For a given experimental watershot data set, a set of measured TOF values, **TOF_m_**, were determined by cross-correlating each trace with a matched filter and taking the position of the resulting maximum peaks. This matched filter was obtained by sampling the wavelet of an ideal, directly transmitted trace when both transducer arrays were placed facing each other. While systematic errors could be introduced in **TOF_m_** by incorrectly selecting the starting time of the matched filter, any constant errors can be absorbed by *τ* when optimised for a constant delay across all traces.

Before starting the element localisation optimisation, the modelled transducer element positions *p* were initialised to nominal positions (a dodecagon arrangement of transducer arrays with a width of 75.00 mm, as illustrated in Fig. 5a), and *τ* was set to 4.00 × 10^−1^ *μ*s. A set of computed TOF values TOF_c_ could then be calculated using eqn (9). Calibrating these transducer positions could then be defined as a non-linear least-squares problem where the goal is to find a set of element positions and *τ*, such that the difference between **TOF_m_** and **TOF_c_** is optimised. This is given by the objective function

(11) $$minF_{\text{p}}(\text{p},\tau )=\left\{\frac{1}{2}∥{\text{TOF}}_{\text{m}}-{\text{TOF}}_{\text{c}}{∥}^{2}\right\}$$

where F_p_ is the residual between the measured and computed TOF sets. A Gauss−Newton algorithm was applied to optimise F_p_ iteratively until reaching a sufficiently low error between these TOF vectors.

Each transducer element was represented as a point on the 2-D imaging plane with a y-coordinate of zero. Therefore, for *P*_e_ transducers, this gave a total of 2*P*_e_ + 1 unknown variables to solve for. Given that the ring array configuration had 3072 transducer elements, this would give 6145 variables to be estimated. However, as these elements lie along rigid transducer arrays, the number of unknowns can be significantly reduced by solving for the position and orientation of the probes instead of individual elements. In three dimensions, these transducer arrays can be considered as lines of elements (element pitch = 2.95 × 10^−1^ mm) with a position given by x, y and z coordinates and an orientation defined by angles *α, β* and *γ* about the x, y and z axes respectively. If we again consider these transducers as lying on a 2-D plane, the *α, β* and *γ* coordinates for each array can be set to zero. For a total of *P*_a_ probe positions, this gives 3*P*_a_ + 1 unknowns. Given that the ring array consists of 32 probe positions, this gives only 97 unknown variables to solve for, which is significantly less than the element-wise optimisation problem. The objective function defined by eqn (11) remains unchanged regardless of whether we solved for array or element positions. While only solving for the relative positions and orientations between transducer arrays, there are infinite solutions. Therefore, to anchor the solution to global coordinates, we set the first probe to the origin with a *β* rotation set to zero. Before running the element localisation method, the mean travel time difference between TOF_m_ and TOF_c_ was 8.79 ± 5.48 × 10^−1^ *μ*s, with a *τ* value of 4.00 × 10^−1^ *μ*s. After 15 iterations of the element localisation method, the mean travel time difference was reduced to 7.20 × 10^−3^ ± 7.98 × 10^−3^ *μ*s, with an optimised *τ* value of 3.46 *μ*s.

### Source wavelet optimization

In this section, we discuss the localisation of the source wavelets of each source used while imaging. These are required when running FWI to model transmission events accurately. The observed watershot data set of each imaging experiment provided valuable information for modelling these source wavelets as these signal data contain measurements of the wavefields transmitted between source−receiver pairs of transducers. We then generated corresponding synthetic watershot data sets by numerically solving the acoustic wave equation using an initial set of predicted source wavelets and the previously calculated modelling parameters c and **p**. We then optimised these source wavelets by applying a Wiener filter to correct for the discrepancies between the resulting synthetic and the observed watershot data sets.

We acquired an initial set of source wavelets by sampling from the traces of the observed watershot data set. Each trace was considered a measurement of the wavefield generated by a transmitting source at the position of a receiver. This trace was therefore assumed to consist only of a wavelet with a travel time proportional to the distance between that source−receiver pair of transducers. The set of travel times for all traces was provided by **TOF_c_**, which was calculated using the distances between the final set of modelled transducer positions **p**. We then applied the normal moveout (NMO) correction method [22] to remove the travel time from each trace, resulting in stacks of coherent wavelets aligned at t = 0 for each source element. Taking the mean of each of these stacks then provides an initial set of source wavelets. We then used these predicted source wavelets to generate a synthetic watershot data set that could be compared with the observed watershot data set. We would expect these data sets to match closely when both the transducer positions, water velocity and source wavelets are sufficiently accurate to model the real watershot wavefields. To correct for any discrepancies between these data sets caused by the modeled source wavelets and transducer positions, we calculated a Wiener filter that maps the NMO sampled wavelets of the synthetic watershot data set from NMO sampled wavelets of the observed watershot data [23]. We then used these corrected source wavelets to generate a second synthetic watershot to confirm that the final source wavelets and transducer element positions were sufficiently accurate to model the real transmit events when running FWI.

### Evaluating full-waveform inversion parameters

If we were to invert the observed watershot data set using the true acoustic modelling parameters with FWI, we would expect the solution to recover a homogeneous acoustic model with SoS values comparable to the true water velocity. Therefore, to evaluate our optimised transducer positions and source wavelet parameters, FWI can be run using experimentally acquired watershot data to determine whether the recovered water SoS values are comparable to the measured water velocity. Examples of this test using the rotary configuration can be seen in Figure 5 for the following cases: (a) with calibrated source wavelets and uncalibrated, nominal transducer array positions, (b) with the uncalibrated source wavelets and the calibrated transducer array positions and (c) with both calibrated source wavelets and transducer array positions. We performed these FWI reconstructions using a maximum frequency of 0.55 MHz for 15 iterations and a water-starting model set to 1481.40 m/s, the experimentally measured water velocity for this acquisition.

Even though there appears to be little visible difference between the nominal and calibrated transducer array positions, the travel time errors introduced when using uncalibrated transducer element positions were sufficient to produce the erroneous SoS model in Figure 5a. The histogram of recovered velocity values for this reconstruction is highly heterogeneous, with a mean SoS of 1466.20 ± 15.48 m/s, significantly lower than the measured water velocity for this experiment. Similarly, we also saw reconstruction errors when running FWI with calibrated transducer positions and uncalibrated source wavelets, as illustrated in Figure 5b. The histogram of recovered velocity values for this result gives a mean SoS of 1445.14 ± 15.07 m/s, which is also significantly lower than the experimentally measured water velocity. This indicates that the calibration of source wavelets is also crucial to run FWI successfully. Conversely, when using calibrated transducer array positions and source wavelets, the resulting water model did not contain significant artefacts and exhibited SoS values that were close to the experimentally measured water velocity. This was supported by the mean water velocity of 1481.28 ± 1.54 m/s and the low variance of SoS values given by the histogram in Figure 5c. These homogeneous recovered water values suggest that the synthetic watershot data closely match the experimentally observed data when using these FWI parameters. Therefore, these parameters can be considered suitable for running FWI using data acquired by the dual-probe USCT acquisition system.

### Full-waveform inversion reconstructions

Full-waveform inversion operations in this study were performed in the time domain using a multifrequency sequence. The lowest frequency at which signal could be distinguished from noise with an SNR of 40 dB when imaging with P4-1 probes was measured to be 0.55 MHz. Unless otherwise stated, each FWI run used this value as a starting frequency. The maximum spatial resolution when using full wave reconstruction methods is of the order of half of the wave-length of the highest frequency in the signal data [7]. By setting the upper-frequency limit of the multifrequency sequence to 1.60 MHz, the upper spatial resolution limit was ~4.62 × 10^−1^ mm. This allowed for the recovery of sub-millimeter features of the brain phantom. The multi-frequency sequence consisted of 22 frequency bands centred on frequencies given by the function y (x) = (2.40 × 10^−4^)(*x* − 1)^2^ + 5.50 × 10^−1^ for frequency step *x*∈ℤ|1≤*x*≤22. This parabolic set of frequencies meant that a large proportion of FWI iterations were performed at the lower range of frequencies so that the solution first recovers a complete low-resolution model of the imaging target before introducing information for finer features at the higher-frequency bands. We ran three FWI iterations for each frequency band, each using a third of all available data, resulting in 66 iterations per FWI run. While inverting for acoustic SoS models, density and acoustic absorption modelling parameters were fixed to constant values for water *ρ* = 1000 kg/m^3^ and *Q* = 1000, respectively. We used a spatial grid spacing of 2.2 × 10^−1^ mm and a time step of 5.00 × 10^−2^ *μ*m for all frequency steps. Forward modelling and FWI operations were performed using the Fullwave software, as described by Warner et al. [7].

## Results

### Linear array configuration

Here we discuss the acquisition of USCT data and FWI results when imaging with the linear array dual-probe configuration. The true SoS model of the fabricated 2.5-D phantom is unknown, but we expect a 2-D cross-section of this phantom to be similar to the numerical brain phantom seen in Figure 4a. This is because the numerical model was used to design the 3-D-printed moulds for fabricating the phantom. The SoS values of the numerical model were derived from measurements of the skull-mimicking layer and PVA brain tissue mimic samples. Using this numerical model, we generated a corresponding synthetic data set for every experimentally acquired data set. As the SoS model and FWI parameters were known for each synthetic data set, these provided the ideal case for each imaging problem.

### Linear array data acquisition

The two linear arrays of this configuration were formed by applying independent 1-D linear motor steps to each probe. We achieved this by using a pair of computer-controlled linear motors (BiSlide, Velmex, Bloomfield, NY, USA) to position each probe to one of five positions on both sides of the imaging target.

**Figure 3:**
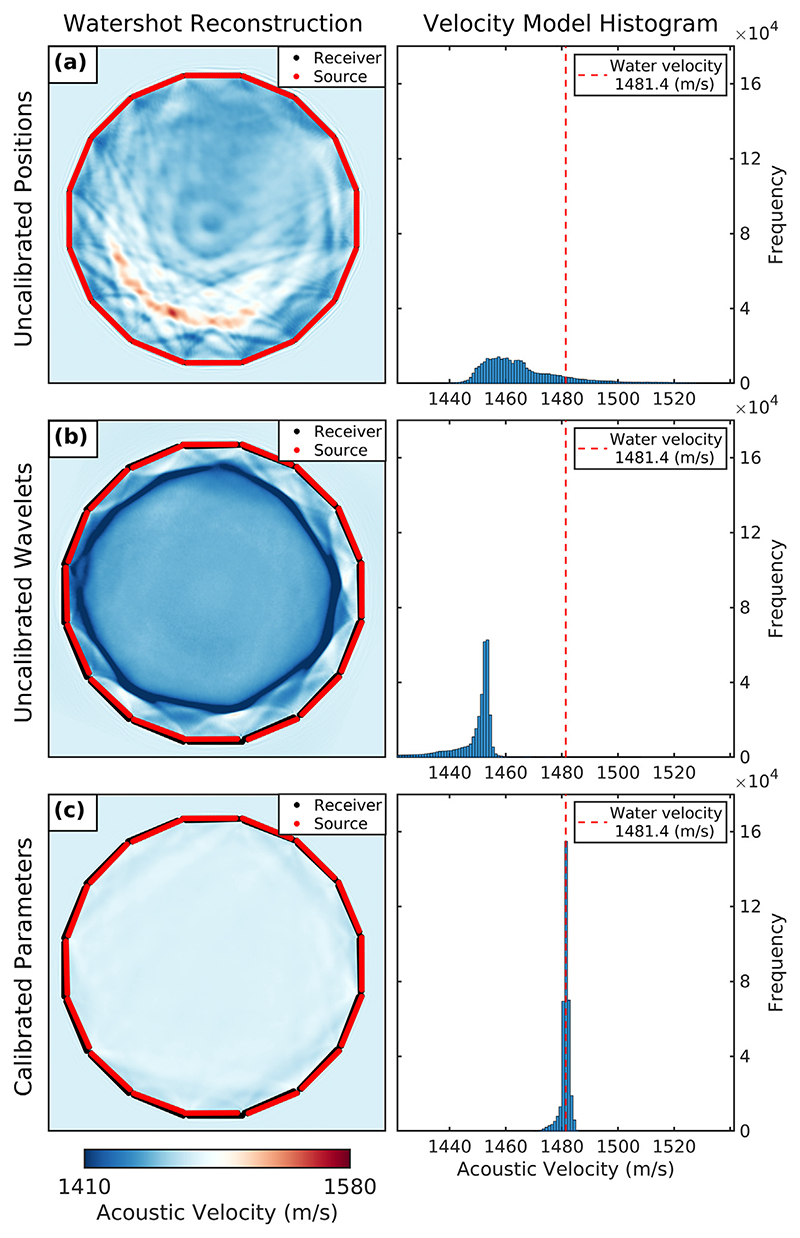
Full-waveform watershot reconstructions for evaluating the calibrated transducer element position and source wavelet parameters. Reconstructions and corresponding histograms of recovered water velocity values are given for inversion while using (a) nominal transducer positions and calibrated source wavelets; (b) calibrated transducer positions and uncalibrated source wavelets; and (c) calibrated transducer positions and source wavelets.

The motor step between positions was equal to the length of a P4-1 aperture (28.32 mm), resulting in two parallel 141.60 mm long transducer arrays, each consisting of 480 elements. The position of the imaging target relative to these two linear arrays can be seen in Figure 3a. A sequence of motor steps was applied to translate and acquire USCT data over all 25 unique source−receiver array positions. All 96 elements of each array position were set as receivers, while a subset of equally spaced 24 elements was selected as sources, providing a total of 240 sources and 960 receivers. However, a drawback of this method is that signals reflected from the imaging target could be acquired only using elements of the transmitting array (as shown by the elements highlighted in *blue* on either side of the source in Figure 3a).

### Linear array brain phantom reconstruction results

To evaluate the performance of the dual-probe linear array system, we performed FWI using both synthetic and experimental USCT data sets. The synthetic data were generated using the numerical brain phantom model and the experimental setup illustrated in Figure 3a. To assess the suitability of the linear array configuration for acquiring USCT imaging data, we first focused on reconstructing the brain model of the phantom without the skull layer. This was because the skull layer was more challenging to reconstruct than the brain model as the SoS difference between the skull layer and water was much greater and it scattered ultrasonic waves more than the brain tissue mimic.

Given that only a small difference in SoS was expected between water (~1484 m/s) and the highest-velocity brain model layer (~1520 m/s), we first attempted to recover the brain phantom using the water-starting model illustrated in Figure 3b. The resulting reconstructions from the synthetic and experimental cases can be seen in Figure 3c and 3d, respectively. The experimental and synthetic results were found to contain comparable, partial reconstructions of the phantom. In both cases, the irregular features of the brain model were recovered, and parts of the surface of the brain model were visible. However, the edges of the phantom facing the transducer arrays were not recovered in either case, and the distributions of SoS values in the brain models were heterogeneous. Given that the SoS values of each brain region of the numerical brain model were homogeneous, this suggested that these recovered SoS values were erroneous. Given the similarities between the synthetic and homogeneous results, the suboptimal brain models were likely caused by the insufficient distribution of transducer positions provided by the linear array configuration. To improve these reconstructions, we then attempted to help the inversion process converge to a more accurate brain model by using the smooth brain starting model shown in Figure 3e. This was created by applying a 2-D Gaussian filter on the numerical brain model. Using this new starting model, we repeated the synthetic and experimental FWI runs to give the recovered SoS brain models seen in Figure 3f and 3g, respectively. These FWI reconstructions had higher SoS values that were more consistent with the true synthetic brain model than the water-starting model FWI results. However, the distribution of these SoS values remained homogeneous. Furthermore, the edges of the phantom facing the transducers were not recovered. The root mean square (RMS) error values for the synthetic results when using the water and smooth brain starting models were 13.56 and 9.25 m/s, respectively relative to the true numerical brain model. This suggested that prior information about the brain model produced a small improvement in the accuracy of the brain model. However, the suboptimal recovered synthetic and experimental brain models when using both starting models suggested that this configuration was poorly suited for USCT imaging as it did not provide enough transducer positions to fully image the brain-mimicking phantom.

### Ring array configuration and ring array data acquisition

The ring array configuration was used to perform 360° scans of the 2.5-D phantom. Both probes were translated to 16 positions about this ring array, each separated by a rotary step size of 22.50°. Each probe was equipped with a separate rotary motor to allow for independent rotations. These motors included a large aperture rotary table (Standa, Vilnius, Lithuania) and a centrally positioned rotary motor (PRMTZ8/M, Thorlabs, Inc., Newton, NJ, US). The radius of rotation for both probes was 75.00 mm to provide a sufficiently large imaging region for the 2.5-D phantom (the number of motor steps and radius could be adjusted for different imaging targets). When setting a source element of a probe to transmit during an imaging sequence, the second probe would then be assigned as the receiver array. This receiver array would then be rotated to measure the transmit event at multiple positions about the ring array. An example set of receiver probe positions for a given source can be seen in Figure 6a. A separation of two probe positions between the source and receiver arrays was maintained to avoid collisions when imaging, giving 11 receiver array positions for every source array position. The source array can also be assigned as a receiver to acquire USCT signal data in reflection (as indicated by the *blue elements* on the source array in Fig. 6a). To perform a scan of an imaging target using this system, we repeated this imaging sequence for all sources over all 16 source array positions on the ring. The resulting ring array configuration is comparable to previously proposed dual-probe USCT imaging systems, such as the multimodal ultrasound breast (MUBI) imaging platform designed by Camacho et al. [24] for imaging the breast using ultrasound reflection tomography. As in the linear array case, we used a subset of 24 sources at each source array position while receiving across all 96 elements at every receiver array position. This gave 384 unique source element positions, each transmitting to a subset of 1152 of 3072 receiver elements.

### Ring array brain phantom reconstruction results

To assess the feasibility of the dual-probe rotary array configuration, FWI was applied to image the brain phantom using both synthetic and experimental USCT data sets. The position of the brain phantom relative to the transducer array can be seen in Figure 6a, and the true synthetic brain model data can be seen in Figure 6b. We obtained high-quality FWI reconstructions of the brain phantom using this configuration in both the synthetic and experimental cases, as illustrated in Figure 6c and 6e, respectively. The full surface, irregular boundaries and central anechoic cavity of the brain phantom are all visible in both reconstructions. Additionally, the synthetic result was composed of distinct, homogeneous regions that match well the true SoS values of the synthetic brain model. This can also be said for the experimental FWI result, albeit with a greater variation of SoS values within each brain layer. However, these variations were likely in part caused by small variations in the acoustic velocity of the real PVA cryogel during the fabrication of the phantom. The experimental FWI reconstruction closely matches the photo of the brain model in Figure 6d, including the slightly collapsed central cavity relative to the true synthetic brain model. The improvement in the quality of these brain models was owing to the large number of evenly distributed transducers placed around the imaging target when using the ring array. This configuration also allowed more reflected signal data to be received, which provided information for reconstructing the complete surface of the imaging target when running FWI. The RMS error between the synthetic FWI result and the numerical synthetic brain model was 2.14 m/s, a significant improvement compared with the previous linear array configuration results. As these reconstructions closely matched the expected velocity model of the brain phantom, it was not necessary to repeat this experiment using a starting model that contained prior information about the brain model.

### Numerical brain and skull phantom reconstruction results

**Figure 4:**
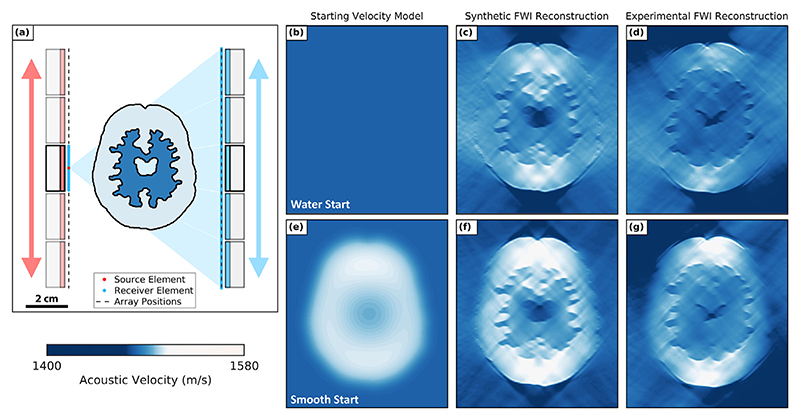
Full-waveform inversion (FWI) results when imaging the brain phantom with the linear array configuration. (a) Dual-probe linear array configuration with five transducer array positions placed on either side of the true numerical brain phantom. (b) Water-starting model. (c) Synthetic FWI reconstruction from the water-starting model. (d) Experimental FWI reconstruction from the water-starting model. (e) Smooth brain starting model. (f) Synthetic FWI reconstruction from the smooth brain starting model. (g) Experimental FWI reconstruction from the smooth brain starting model.

**Figure 5:**
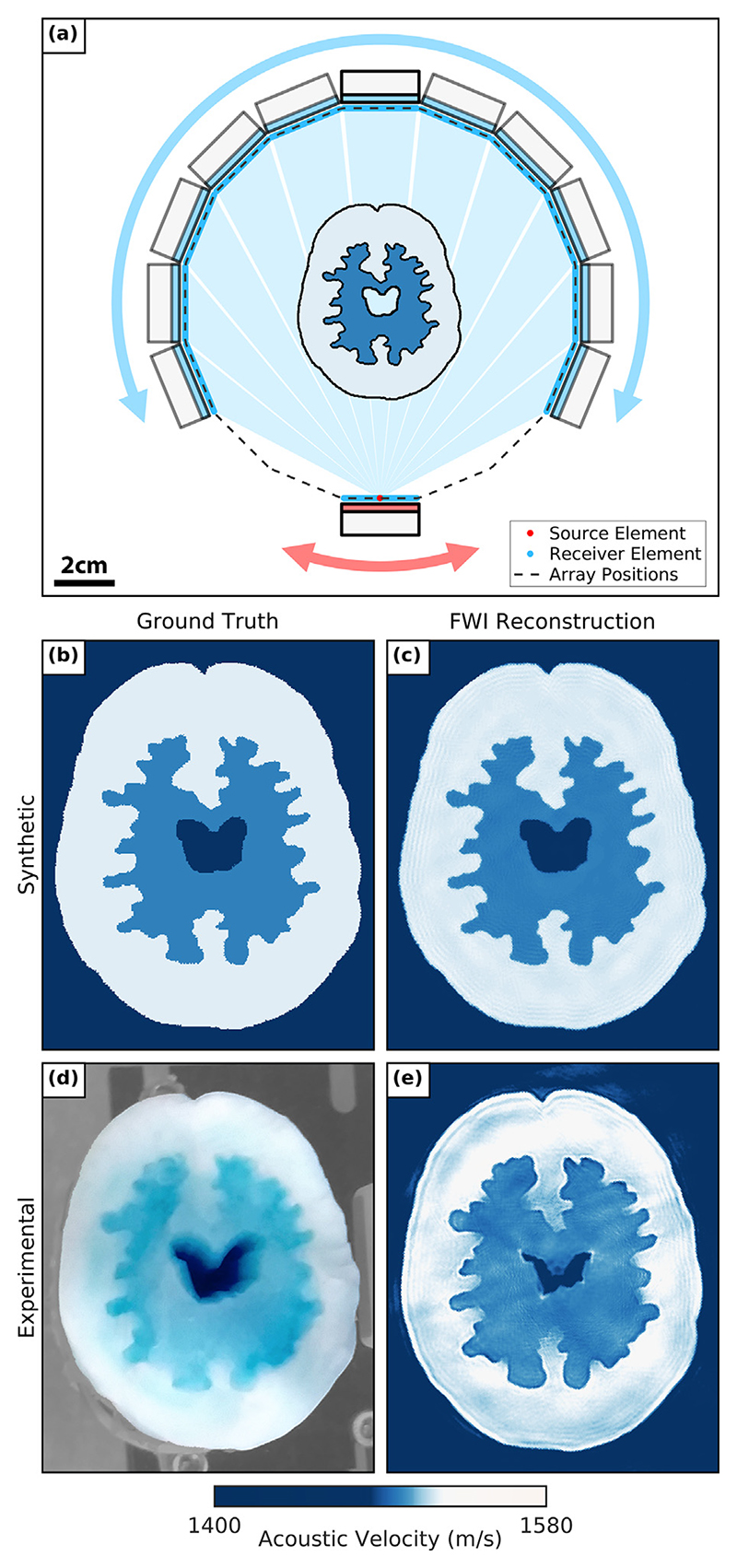
Full-waveform inversion (FWI) results when imaging the brain phantom with the rotary array configuration. (a) Dual-probe rotary array configuration of 16 transducer positions with the numerical brain phantom model. (b) True numerical brain phantom model. (c) Synthetic FWI reconstruction of the brain phantom. (d) Overhead photo of the brain phantom while being imaged. (e) Experimental FWI reconstruction of the brain phantom.

After the promising FWI brain model reconstructions when using the ring array configuration, we repeated the ring array scan of the brain model with the 3-D-printed skull layer in place. The numerical model for this imaging problem can be seen in Figure 4a.

The introduction of the high-velocity skull layer (~2550 m/s) created additional acoustic events that were encoded in the resulting signal data. Furthermore, the skull layer increased the travel-time differences between the USCT data sets when only water was present and when the 2.5-D phantom was imaged. We demonstrate this by taking sample traces for the source−receiver pair indicated in Figure 4a to compare both imaging data sets, which can be seen plotted in Figure 4b and 4c, respectively. The sampled trace from imaging the 2.5-D phantom arrived 1.85 *μ*s faster than the sampled watershot trace, which was owing to the high SoS value of the skull layer. As this phase shift was greater than 90° at the lowest frequency band for these data sets, we would expect inversions of the 2.5-D phantom from a water-starting model to cycle-skip. We demonstrated this by running FWI using a synthetic data set generated for the numerical brain and skull model given in Figure 4a with the water-starting model shown in Figure 4f. This produced the unsuccessful FWI reconstruction illustrated in Figure 4g. The skull layer was not recovered during this inversion, and the SoS values of the brain model were erroneous. The RMS error between this recovered brain model and the true numerical phantom was 37.06 m/s.

### Matched reflection skull surface modelling

The FWI reconstruction of the 2.5-D phantom from a water-starting model was not successful, as was expected because of the significant travel-time differences between the skull layer and water. However, the skull layer surface was visible in the FWI reconstruction in Figure 4g. This was owing to the strong reflections collected by the ring array from the skull layer. From this observation, we developed an iterative signal-matching algorithm to identify a set of points representing the surface of the skull layer. This algorithm was designed to update the positions of the surface points such that the misfit between the observed and synthetic reflection travel times was minimised. For this method, only reflections directly received by the same transducer that transmitted were considered, giving 384 reflection traces for all sources of the ring array. The optimised predicted skull surface from this algorithm was then used to design starting models with prior information about the skull layer to mitigate cycle skipping.

The reflection matching problem is presented in Figure 7a, which illustrates the position of the transducer elements relative to the skull layer surface (in *black*), the starting predicted skull surface (in *blue*) and the final predicted skull surface (in *red*) after 10 iterations of the surface localisation algorithm. Additionally, a signal plot is given to compare the observed and synthetic reflections acquired when transmitting and receiving from Source 16 in Figure 7b. A large travel time difference Δ*t* was observed in this signal plot between the synthetic reflection from the input skull layer (Predicted Surface 01) and the observed reflection. The earlier arrival of the synthetic reflection meant that the predicted surface needed to be displaced further from the source by a distance proportional to Δ*t*. Given the measured water velocity *c*, the new surface distance from the source is given by *r* = *d* + Δ*d*, where d is the current distance of the nearest surface point to the source, and Δ*d* is the estimated required position update given by Δ*d* = *c*⋅Δ*t*. Performing these surface point updates was achieved by calculating velocity fields to displace surface points towards positions *r* away from each source. To ensure the surface point updates from each source were applied only to the closest current surface points to each source, the velocity fields were constrained to be zero everywhere but the neighbourhood of these closest points. The magnitudes of these velocity fields were proportional to Δ*d* calculated for each source. This was implemented to introduce finer surface point updates as the differences between the synthetic and observed travel times approached zero after consecutive model updates. The mean of all calculated velocity fields for the current predicted surface was then taken and applied to the current set of surface points for 100 time steps to provide the next predicted surface. This was repeated for either a fixed number of iterations or until the displacements applied to the surface points became negligibly small. After running the iterative signal-matching algorithm for 10 iterations, we found that the final set of synthetic reflections closely matched the observed reflection data, as demonstrated by comparing the *black and red* signal plots for Source 16 in Figure 7. The mean travel time difference between the first and final iterations was 9.98 ± 1.48 and 1.51 × 10^−1^ ± 5.66 × 10^−1^ *μ*s, respectively, which indicated that the final predicted surface reflection data set matched the experimental reflection data set more closely than the starting predicted surface data set. Additionally, the recovered outline of the skull layer in *red* in Figure 4f was morphologically similar to the numerical skull layer model in Figure 2.

### Predicted skull starting model

Using the skull layer surface model recovered from the reflection matching algorithm, we then attempted to design a starting model that would improve the synthetic brain and skull FWI reconstruction while using minimal prior information about the true skull and brain phantom. In a clinical setting, the skull surface and relative position to the transducers could be recovered using an approach similar to the reflection matching method proposed in this study employing the reflection data from a full-head USCT data set acquired to perform FWI. Without any prior information on the inner surface of the skull layer, this skull model was given a constant thickness determined by the expected mean skull thickness. Measurements of the skull layer model gave a mean skull thickness of 2.24 ± 0.48 mm. The predicted skull starting model consisted of this skull layer with a smooth brain model, as illustrated in Figure 4h.

To demonstrate the effectiveness of this starting model in mitigating cycle skipping, we compared traces sampled from the experimental 2.5-D USCT data set and a synthetic data set of the predicted skull starting model, which are both plotted in Figure 4c and 4d. The travel times of these sampled traces match closely. This suggests that using this starting model would likely lead to fewer cycle skipping-related issues when running FWI using this starting model compared with a water-starting model. To test this, we attempted a synthetic FWI reconstruction of the numerical skull and brain acoustic model using the predicted starting model. The final iteration of this inversion can be seen in Figure 4i. In this FWI reconstruction, some recognisable features of the brain model were recovered, such as the water-filled cavity. However, the predicted skull layer was not significantly updated during the inversion. The difference between the predicted and real skull models resulted in artefacts and erroneous SoS values in the brain model. The RMS error between the synthetic brain model reconstruction and the numerical brain model was 8.45 m/s. These observations suggest that introducing this simple predicted starting model allowed the inversion to recover an acoustic model that more closely resembled the true SoS brain model compared with the water-starting model reconstruction.

### Fitted skull starting model

To create a more accurate starting model, we created the fitted skull starting model illustrated in Figure 4j. This starting model consisted of the true numerical skull model fitted to the skull layer recovered from the reflection matching algorithm with a smooth brain model. Sample traces taken from both the observed 2.5-D USCT data set and a synthetic data set using the fitted skull starting model are plotted in Figure 4c and 4e. These traces are in phase, which suggests that the fitted starting model is a good representation of the real imaging problem. The synthetic FWI reconstruction using the fitted skull starting model can be seen in Figure 4k. This reconstruction resembled the true numerical model more closely than previous reconstructions and had fine features such as the irregular boundaries between the brain layers and central water-filled cavity. Furthermore, the RMS error between this reconstructed brain model and the true numerical brain model was 3.35 m/s. This reduced error supported the observation that this result was an improvement on previous reconstructions for this synthetic brain and skull imaging problem.

### Experimental 2.5-D brain and skull phantom reconstruction results

**Figure 6:**
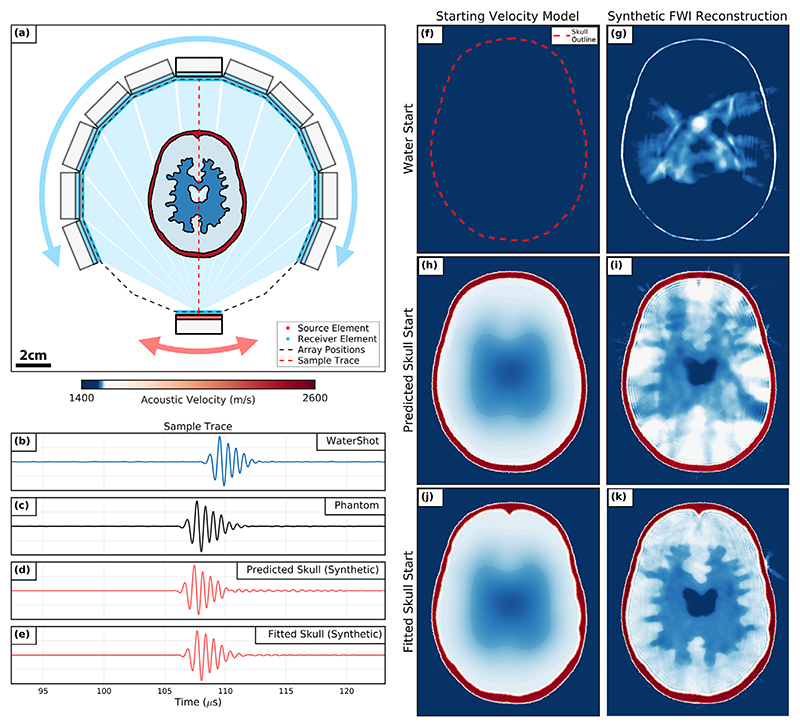
Synthetic full-waveform inversion (FWI) results when imaging the 2.5-D phantom using the rotary array configuration. (a) Experimental setup of the rotary acquisition indicating the transducer positions for a shot gather when transmitting from the first source array. (b−e) Sample traces taken when transmitting through water, the 2.5-D phantom, the predicted skull starting model and the fitted skull starting model. (f, g) Water starting model and reconstruction with the skull outline plotted in *red*. (h, i) Predicted skull starting model and reconstruction. (j, k) Fitted skull starting model and reconstruction.

**Figure 7:**
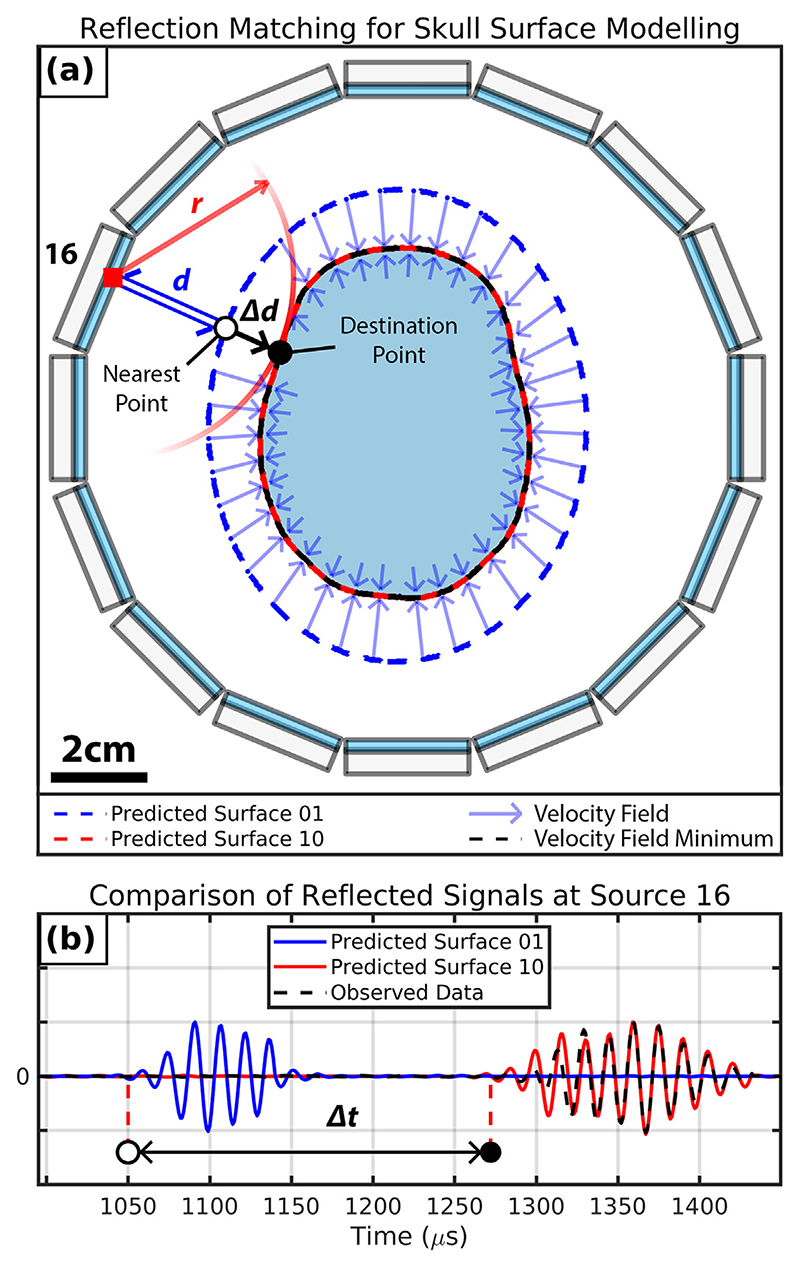
Matching reflected signals to model the surface of the skull. (a) A polygon representing the brain and skull phantom relative to different predicted skull surfaces and the rotary configuration of transducer arrays. *d* represents the distance between Source 16 and the nearest point of the latest predicted skull surface. Δ*d* represents the additional distance this nearest point should be displaced to be at *r*, the distance that would minimize the travel time difference between the observed and synthetic signals acquired by Source 16. This update is applied to surface points along the ray from Source 16 towards the nearest point on the current predicted skull surface. (b) Reflection signal plot comparing the synthetic reflection signals from the predicted skull surfaces at iterations 01 and 10 plotted to the observed experimental reflection signal.

**Figure 8:**
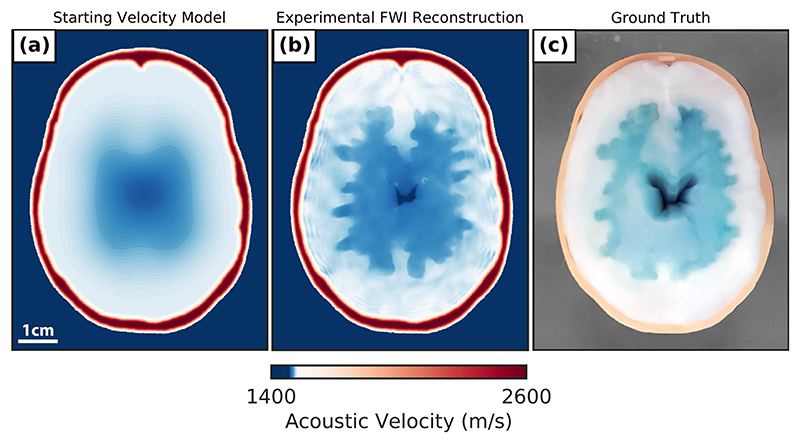
Full-waveform inversion (FWI) results using an experimental dataset from imaging the 2.5-D phantom with the rotary array configuration. (a) The fitted skull starting model, (b) Experimental FWI reconstruction of the brain and skull phantom. (c) Overhead ground truth photo of the brain and skull phantom while being imaged.

Given the promising synthetic 2.5-D phantom reconstruction results when using the fitted skull starting model, the same method was used to recover a SoS model of the 2.5-D brain and skull phantom from an experimentally acquired USCT data set. This inversion was performed using the fitted skull starting model shown in Figure 8a. A photograph of the 2.5-D phantom can be seen in Figure 8c. The real 2.5-D phantom can be seen to match closely the numerical brain and skull model. However, small differences are apparent in the fabricated 2.5-D phantom because of the expansion of the PVA brain tissue mimic during the freeze−thaw cycles. This is most pronounced when observing the reduced size of the central anechoic cavity. The experimental FWI reconstruction seen in Figure 8b has a recovered brain model with layers that closely match those seen in the photographs of the real brain model given in Figures 6d and 8c. Furthermore, the mean SoS values from random samples of the different regions of this recovered brain model were 1478.45 ± 1.01, 1501.79 ± 1.01 and 1521.85 ± 4.29 m/s for the water-filled central cavity, inner brain layer and outer brain layer, respectively. These results indicate that the recovered acoustic model closely resembles the true numerical brain and skull model. The measured SoS values for the different regions of the brain model match closely the expected values for the PVA brain tissue mimic and water, indicating that the model is a successful reconstruction of the brain after imaging through the skull-mimicking layer. However, there is a slight reduction in contrast between the layers of the brain phantom compared with the recovered brain model when the skull was not present. This is likely owing to small variations in the shape, SoS values and position of the true skull model compared with the fitted skull starting model used for this FWI reconstruction.

## Discussion

**Figure 9:**
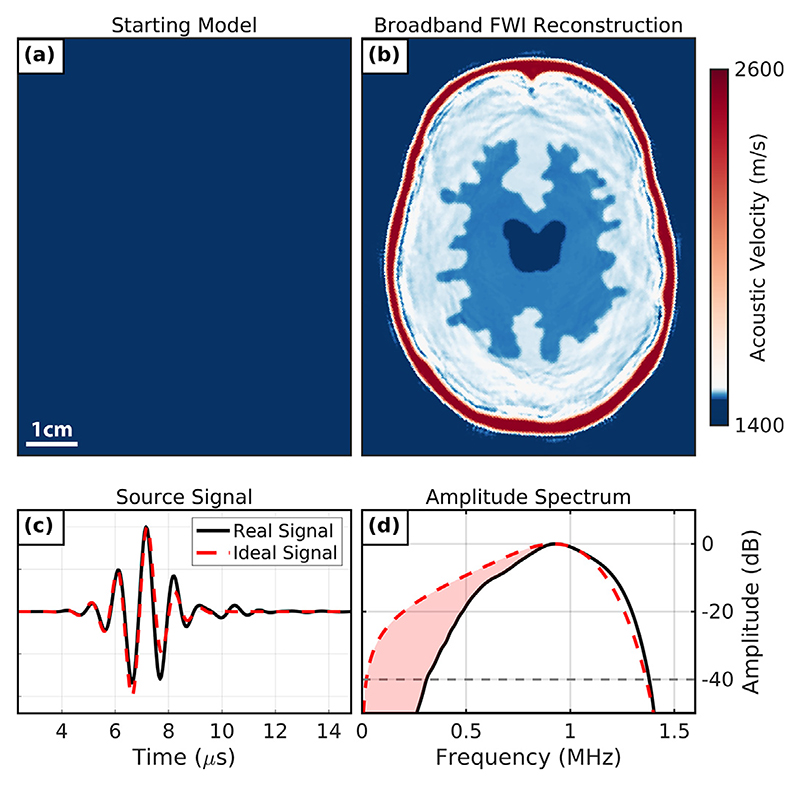
An ideal synthetic full-waveform inversion (FWI) reconstruction from water with broadband data. (a) Starting model of homogeneous water. (b) Ideal broadband full-waveform reconstruction. (c) The real and ideal broadband source signals. (d) Amplitude spectrum of the real and ideal broadband source signal. Highlighted in *red* is the low-frequency content difference between these spectra.

The proposed USCT imaging methodology using the rotary configuration shows promise as a research tool for further exploring novel imaging applications of FWI, particularly for soft tissue imaging problems, such as imaging breast tissue. However, FWI from the lowest possible starting frequency of 0.55 MHz when using the P4-1 probes was insufficient for recovering high-SoS skull-mimicking material from water. This was illustrated by the failed reconstruction of the 2.5-D brain and skull phantom from water in Figure 4g. For this reason, conventional off-the-shelf medical ultrasound devices were found to be poorly suited for FWI imaging. To overcome this problem, a fitted skull starting model (Fig. 4j) was used to successfully recover the brain model of the 2.5-D phantom using FWI. However, this method required a significant amount of prior information about the phantom, including the location, morphology and SoS of the 3-D-printed resin skull-mimicking layer. This method would not be practical in a clinical setting as this prior information may not be immediately available and an alternative imaging modality would be required to provide the skull model. Relying on an alternative imaging modality for this purpose would increase the running costs required to recover an acoustic brain model and so would reduce the impact of using FWI as a neuroimaging modality. Alternatively, imaging could be performed using transducers that can transmit and receive at sufficiently low frequencies to mitigate the impact of cycle-skipping. We demonstrated this by repeating the synthetic brain and skull phantom imaging problem after replacing the P4-1 source signals with the ideal broadband source wavelet plotted in Figure 9b. As indicated by the amplitude spectrum plotted in Figure 9c, this broad-band signal has a much lower 40 dB limit of 0.07 MHz compared with the P4-1 signal. FWI was then run with this ideal broadband USCT data while using a lower starting frequency of 0.15 MHz and the water-starting model in Figure 9a (multifrequency sequence from 0.15 to 1.60 MHz over 25 frequency steps, total number of iterations = 75). As seen in the final iteration of this inversion given in Figure 9b, when inverting with broadband signal data, it was possible to successfully recover both the brain model and the skull layer of the 2.5-D phantom without providing any prior information about the imaging target. This suggests that one avenue of investigation to improve the dual-probe USCT acquisition system would be to explore the fabrication of broadband ultrasound transducers with bandwidths closer to that of the ideal signal given in Figure 9.

It was also observed that the inversion process did not successfully update the predicted skull starting model (Fig. 4h) to recover a more accurate skull model. This occurred even though the numerical skull consisted of a constant SoS value without any microstructure. This suggests that running FWI from a starting frequency of ~0.55 MHz may not be sufficient to update the skull to account for the impact of microstructure on the propagation of ultrasound waves. To address this, further work is required to investigate the impact of microstructure when running FWI. This could be carried out by repeating the imaging experiments discussed in this study using either more detailed fabricated skull models with microstructure or by surrounding an ultrasound brain phantom with ex vivo skull tissue.

While the P4-1 USCT data sets were found to have insufficient low-frequency signal content for imaging the skull model with FWI, the P4-1 signal data have an upper-frequency limit of ~3.50 MHz. This suggests that these probes may be better suited for high-resolution imaging problems with smaller imaging targets. Consequently, the dual-probe system may have potential as a non-invasive, high-resolution neuroimaging tool for small animal models. In these cases, the thinner skulls and reduced head size of these animal models would result in USCT imaging data with smaller phase shifts with respect to corresponding water shots, thereby relaxing the lowest frequency required to meet the 90º phase shift criterion. If the resulting phase shifts are found to be sufficiently small to avoid cycle skipping when imaging with P4-1 probes then it may be possible to recover high-resolution head models of small animals. Further investigation is required using ex vivo small animal skull and brain tissue samples to explore the potential of the dual-probe acquisition system for this application.

The proposed USCT acquisition system is ideally suited for research applications that demand a versatile imaging system that can be adapted to various use cases. This system allows for the implementation of different probe configurations and imaging sequences, accommodating a broad range of potential imaging targets for different USCT projects. Furthermore, by replacing the P4-1 probes with 2-D arrays, the imaging system can be applied to 3-D imaging tasks. This could be used to overcome out-of-plane effects when reconstructing complex 3-D geometries with FWI. However, for the system to be effectively employed for FWI brain imaging in adults within a clinical setting, the dual probes would ideally need to be replaced by a single fixed hemispherical array of ultrasound transducers. This is because the current use of rotating ultrasound probes would require longer scanning times compared with fixed arrays, making it challenging to acquire full 3-D data set scans needed to image a patient’s head *in* vivo. Maintaining stillness during the scan could cause discomfort, and any movement by the patient would result in motion artefacts. In comparison, imaging with a fixed hemispherical array would allow full 3-D USCT data sets to be acquired for each transmission, allowing for significantly quicker scan times to mitigate the discomfort to patients while imaging. These data sets can then be used to recover 3-D acoustic models of the brain using the FWI reconstruction methods discussed in this study.

The clinical adoption of FWI ultrasound tomography holds the potential to improve healthcare outcomes by offering a more accessible modality for non-invasive, quantitative, full-brain imaging. This is owing to the inherent affordability and portability of ultrasound imaging hardware when compared with existing gold-standard neuroimaging technologies such as CT and MRI. Furthermore, ultrasound imaging does not require shielding from the potentially harmful ionising radiation and magnetic fields associated with CT and MRI, making it a safer and more cost-effective solution with respect to both infrastructure and operational expenses. These advantages would make an ultrasound tomography neuroimaging solution an ideal option for remote medical centres, which may lack the resources to house existing neuroimaging devices. One promising clinical application of this modality is in diagnosing and measuring brain tumours. Much like conventional tomographic methods, SoS maps of the brain offer insights into the physical properties of brain tissue. These recovered SoS values can be used to distinguish between different types of soft brain tissues and detect anomalous, potentially cancerous tissue. Additionally, the structural information provided by the recovered brain models can aid in the localisation and segmentation of brain tumours. The high portability of ultrasound devices could also allow for point-of-care neuroimaging to be performed in ambulances. This application could have a significant impact on the management of time-sensitive medical conditions, such as stroke, where quick diagnosis and initiation of treatment are crucial for optimal patient outcomes. To administer the appropriate treatment, clinicians must first establish whether the patient has had an ischemic stroke, typically caused by a blood clot, or a hemorrhagic stroke, resulting from bleeding into the brain tissue caused by ruptured blood vessels. Currently, the extent of brain damage and the cause of strokes are established by CT or MRI scans as soon as possible. However, this could potentially be achieved by detecting signs of brain haemorrhages in acoustic brain models recovered using FWI by comparing the SoS of brain tissue permeated with blood with that of healthy brain tissue. The acquisition of this crucial diagnostic information while patients are in transit could allow for the immediate administration of the correct treatment upon their arrival for emergency care. Reducing the interval between the onset of a stroke and the initiation of treatment could increase survival rates and lead to more favourable recovery outcomes.

## Conclusion

In this study, we determined the feasibility of using FWI to perform high-resolution, quantitative imaging of an ultrasound brain and skull-mimicking phantom. We fabricated a 2.5-D brain phantom surrounded by a 3-D-printed skull-mimicking layer to simulate a clinical neuroimaging problem. A low-cost, dual-probe USCT acquisition system was designed using readily available components, and a robust calibration methodology was implemented to enable high-quality, high-resolution FWI reconstructions of the brain and skull phantom from experimentally acquired data. Our results indicated that USCT data acquired using a rotary configuration of the dual-probe acquisition system were most effective for reconstructing the brain and skull phantom. High-quality reconstructions of the brain tissue mimic were achieved without prior information about the imaging target when the skull-mimicking layer was present. Imaging the brain phantom inside the skull was achieved by using a starting model with an accurate representation of the skull layer positioned using a reflection matching algorithm. We found that this approach could be used to successfully reconstruct brain-mimicking tissue from both experimental and synthetic USCT data. The dual-probe system may have potential as a non-invasive, high-resolution neuroimaging tool for small animal models. Further investigation is required using ex vivo small animal skull and brain tissue samples to explore the potential of the dual-probe acquisition system for this application. Overall, this study provides a promising step towards the development of a non-invasive, low-cost and safe neuroimaging method for adults.

## Supplementary Material

Appendix

## Figures and Tables

**Figure 10:**
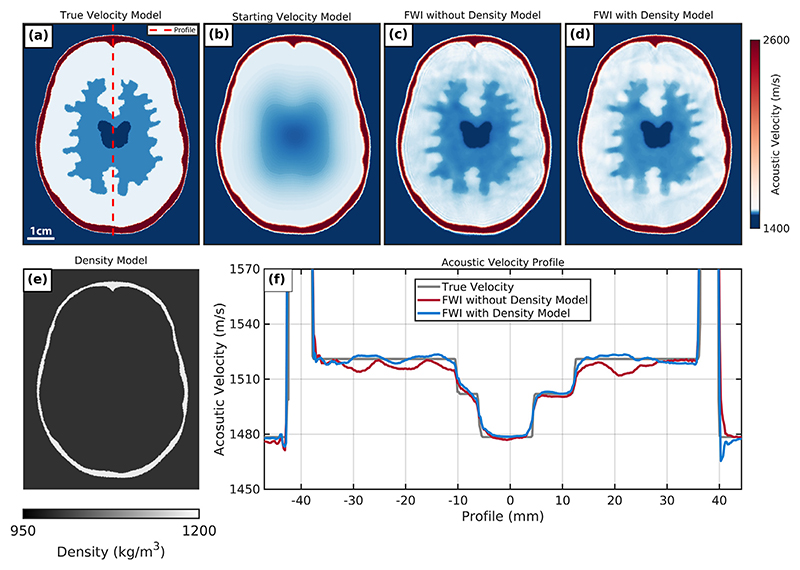
Synthetic full-waveform inversion (FWI) results when imaging the 2.5-D phantom using the rotary array configuration with respect to both speed of sound and density. (a) True numerical speed-of-sound brain and skull model. (b) Fitted skull speed-of-sound starting model. (c) Synthetic FWI reconstruction without true skull density model. (d) Synthetic FWI reconstruction with true skull density model. (e) Fitted skull true density model. (f) Acoustic velocity profile comparing speed-of-sound values sampled from the FWI reconstructions and the true numerical model.

**Table 1:** Speed of sound values of tissue and tissue-mimicking materials

Material	Speed of sound (m/s)
Brain	
Gray matter	1500
White matter	1552 ± 29
Skull	
Cancellous	2118 ± 289
Cortical	2814 ± 337
Araldite 1302	3008 ± 220
Vero Black	2495 ± 8
Formlabs clear resin	2545 ± 11
PVA brain layer	
Outer	1521 ± 3
Inner	1502 ± 4

Tissue properties were sourced from the IT’IS Foundation tissue properties database, Version 4.0 [13]. Measurements for the speed of sound of Araldite 1302 were provided by Robertson et al. [15]. The PVA brain layers and Formlabs clear resin were measured experimentally. PVA, polyvinyl alcohol.

## Data Availability

Data sets related to this article can be found at https://doi.org/10.17632/rbx3ybd5zx.1, an open-source online data repository hosted at Mendeley Data [25].
