# Supplementary material for: Dual-Probe Transcranial Full-Waveform Inversion: A Brain Phantom Feasibility Study
Source: Ultrasound Med Biol. Author manuscript; Available in PMC 2024 Aug 21. (PMC7616382; doi:10.1016/j.ultrasmedbio.2023.06.001)
Supplement: Appendix [file EMS198058-supplement-Appendix.pdf]

Appendix. Impact of density on numerical brain and skull reconstruction

The synthetic USCT data set for the brain and skull imaging problem in Figure 4 was generated using a forward model with respect to the SoS model of the numerical brain and skull phantom. However, in real experimental acquisitions, the density of the skull layer is another physical property that can have an impact on the propagation of acoustic waves. To demonstrate the impact of running FWI without accounting for the density of the skull-mimicking layer, we generated a new synthetic USCT data set with respect to both the numerical skull SoS and density models seen in Figure 10a and 10e. This skull density model consisted of the fitted skull with a density value of 1178 kg/m^3 [17]. We then ran FWI using the updated USCT data set and the SoS starting model seen in Figure 10b for two different cases: in the first case, FWI was run with a constant density model of $\rho = 1000$ kg/m^3, while in the second case, FWI was run with the true skull density model. The FWI reconstructions for these cases can be seen in Figure 10c and 10d, respectively. Both reconstructions closely match the true numerical brain and skull model; however, the reconstruction using the true skull density model had slightly higher brain model SoS values that seem more comparable to those of the numerical brain model. The RMS error between the recovered and true brain models were 4.098 and 3.254 m/s for the inversions without and with the true skull density model, respectively. This illustrates that the FWI reconstruction of the brain that was run with the true density model was more closely matched with the numerical brain model. Furthermore, the acoustic velocity profile comparing these FWI reconstructions can be seen plotted in Figure 10f. Here, the SoS values along the *dotted line* in Figure 10a were plotted for both FWI reconstructions and the true numerical model. This plot illustrates that the SoS values sampled from the FWI reconstruction with the true skull density model matched the true numerical model more closely. These findings suggested that the brain and skull FWI reconstructions may be improved by running with both the fitted skull SoS starting model and a fitted skull density model. Despite this, neglecting the impact of density did not prevent the recovery of a high-quality SoS brain model for the imaging problem considered in this study. However, real skull tissue has a measured mean density of $\rho = 1908$ kg/m^3 [13], which is much greater than that of the skull layer skull-mimicking material. Therefore, the impact of skull tissue density is likely to be far greater when performing transcranial imaging in vivo. For this reason, investigating the impact of neglecting density should be considered in future FWI brain imaging investigations when real skull tissue is present.
